# Robustness and Backbone Motif of a Cancer Network Regulated by miR-17-92 Cluster during the G1/S Transition

**DOI:** 10.1371/journal.pone.0057009

**Published:** 2013-03-01

**Authors:** Lijian Yang, Yan Meng, Chun Bao, Wangheng Liu, Chengzhang Ma, Anbang Li, Zhan Xuan, Ge Shan, Ya Jia

**Affiliations:** 1 Institute of Biophysics and Department of Physics, Central China Normal University, Wuhan, China; 2 School of Life Sciences, University of Science and Technology of China, Hefei, China; Semmelweis University, Hungary

## Abstract

Based on interactions among transcription factors, oncogenes, tumor suppressors and microRNAs, a Boolean model of cancer network regulated by miR-17-92 cluster is constructed, and the network is associated with the control of G1/S transition in the mammalian cell cycle. The robustness properties of this regulatory network are investigated by virtue of the Boolean network theory. It is found that, during G1/S transition in the cell cycle process, the regulatory networks are robustly constructed, and the robustness property is largely preserved with respect to small perturbations to the network. By using the unique process-based approach, the structure of this network is analyzed. It is shown that the network can be decomposed into a backbone motif which provides the main biological functions, and a remaining motif which makes the regulatory system more stable. The critical role of miR-17-92 in suppressing the G1/S cell cycle checkpoint and increasing the uncontrolled proliferation of the cancer cells by targeting a genetic network of interacting proteins is displayed with our model.

## Introduction

Revealing the relationship between structure and function is a central theme in systems biology. The functions of biological network can be explored through mathematical modeling and computational simulation if the biochemical details of the molecular network are known. It is also important to know how network structures contribute to the biological functions. Despite the inevitable existence of external and internal perturbations, such as gene mutation, transcription/translation noise, interaction deletion/addition, and external environmental stimuli, the biological system can usually maintain its functions by changing the steady state or the expression of related genes. Such robustness has been widely observed in many biological systems and events, e.g., chemotaxis in bacteria, immune system, cancers and cell cycle [1]–[4].

It is well known that proliferation of eukaryotic cells is an ordered, tightly regulated process that consists of four phases: G1, S, G2, and M (i.e. G1 → S → G2 → M → G1) [5], [6]. Although cell cycle progression normally relies on stimulation by mitogens and can be blocked by anti-proliferative cytokines, cancer cells abandon these controls and tend to remain in cell cycle [7]. The cells that progress through the cell cycle unchecked may eventually form malignant tumors.

By virtue of Boolean network theory, previous researches robustly constructed various cell cycle regulatory networks [8]–[10]. Most of the initial states in state space of these Boolean networks flow to the biological steady states in cell cycle process of budding yeast (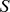. 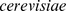) [8], fission yeast (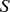. 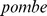) [9], and mammalian cells [10]. A more recent study demonstrated that the cell cycle network structures of both 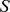. 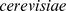 and 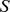. 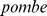 cells can be decomposed into a backbone motif and a remaining motif by using the unique process-based approach [11], where the backbone motif carried out the main biological functionality of cell cycle network.

On the other hand, tremendous growth of our understanding of microRNAs (miRNAs) suggests that miRNAs are involved in the regulation of the cell cycle program of normal and cancer cells [12]. miRNAs are endogenous small non-coding single-stranded RNA, 19 to 23 nucleotides in length. They can inhibit gene expression via binding to its partially complementary sequences within the 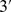 untranslated region of its target mRNAs [13]. Profiling of miRNAs in human cancer specimens and cell lines has revealed a growing number of oncogenic and tumor suppressive miRNAs, among which one of the best known miRNAs is miR-17-92 cluster [14]. Over-expression of the miR-17-92 locus has been identified in a broad range of cancers [15], such as lung cancers, chronic myeloid leukemias, B-cell and mantle cell lymphomas, and hepatocellular tumors. In addition, the miR-17-92 cluster appears to act as a tumor suppressor in some breast and ovarian cancer cell lines [16]. The close relationship between miR-17-92 and cancers indicates that miR-17-92 may regulate fundamental biological processes.

During the cell cycle process, multiple checkpoints are involved to assess extracellular growth signals, cell size, and DNA integrity [17]. Two main checkpoints exist: the G1/S checkpoint and the G2/M checkpoint. G1/S transition is a rate-limiting step and is also known as the restriction point in the cell cycle. After achieving an appropriate cell size, early G1 cells irreversibly cross the checkpoint into the late G1 phase and are committed to undergoing DNA replication (S phase) followed by mitosis [18]. Alterations in components regulating checkpoint traversal and S-phase entry appear to influence the level of tumor cell proliferation. Now the question is whether the robustness properties of cancer regulatory network structure can be ensured at the checkpoints of cell cycle process. Does the miR-17-92 cluster play a crucial role in the cell cycle process? Is there a backbone network that can carry out the biological process?

In this paper we have constructed a cell cycle network to investigate the robustness of this network and the importance of miR-17-92 cluster in the cell cycle process. The network is associated with the control of G1/S transition in the mammalian cell cycle [17]–[19]. Boolean network theory is applied to investigate the robustness properties of this regulatory network. It is shown that, even during the G1/S transition in the cell cycle process, the regulatory network is still robustly constructed. Finally, by using the unique process-based approach [11], we found that the network structure can be decomposed into a backbone motif which provides the main biological functions and a remaining motif which makes the regulatory system more stable.

## Model and Results

### 1. Model of the Cancer Regulatory Network

Some key regulators are involved into the G1/S transition, for examples, the transcription factors E2F and Myc, the oncogenes Cdk2/Cyclin E, Cdc25A and Cdk4/cyclin D, and the tumor suppressors pRb and p27. These regulators constitute a so called cancer network [19]–[21]. Tumor suppressors act to maintain checkpoints, whereas oncogenes allow for checkpoints to be overcome. The transcription factors E2F and Myc, as oncogenes or tumor suppressors (depending on their expression levels), are inhibited by miR-17-92 cluster (which give rises to seven mature microRNAs, including miR-17-5p, miR-17-3p, miR-18a, miR-19a, miR-19b, miR-20, and miR-92-1) [19], [22]. In return, E2F and Myc induce the transcription of miR-17-92, thus forming a negative feedback loop in the interaction network. As one of the first reported and well studied oncomiRs, human miR-17-92 is able to act as both an oncogene and a tumor suppressor in different cellular context. However, the underlying mechanism of either being tumor suppressive or oncogenic for miR-17-92 miRNAs remains unknown.

**Figure 1:**
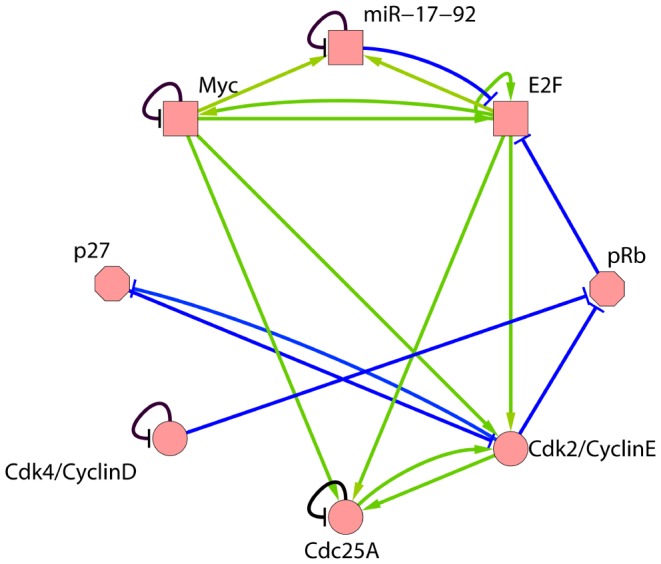
Mammalian cancer cell network during G1/s transition (MGSTR network). The 8-node network is constructed on the basis of previous experimental results [17]–[22]. The circular nodes represent oncogene, the octagon nodes represent tumor suppressors, and the quadrilateral nodes represent oncogenes or tumor suppressors. Green arrow represents active interactions, and the blue (or black) hammerheads represent inhibitory interactions.

Basing on the interactions between the regulatory factors and the miRNAs [19], we have constructed a Boolean model of the mammalian G1/S transition regulatory network (MGSTR network) involving oncogenes, tumor suppressor genes, and miR-17-92, as shown in Fig. 1. This structure contains eight nodes (each node represents a regulatory element) and seventeen lines (each line represents an interaction between nodes). There is often a threshold for the functional copy number of individual molecule in biochemical reactions. Copy number of the gene products higher or lower than the threshold can be represented by two different states: on or off. Therefore, the expression of genes can be considered as a total-or-nothing process, that is, a binary switch which has only two states 1 and 0.

Applying the Boolean theory to our MGSTR network, node 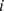 in the network has two states: 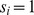 if expressing and 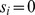 otherwise. Nodes interact and update their states according to the following Boolean functions:
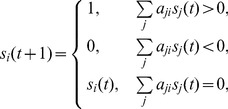
(1)where 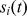 denotes the state of node 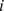 (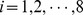) at time 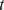. The network structure parameter 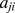 is a 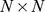 matrix; 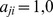, or 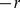 represents respectively the activation, no interaction or inhibition between biological molecules. The self-inhibition (or degradation) effects of these nodes are indicated by black hammerheads in Fig. 1, and we use 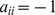 for this kind of self-inhibition. All structure parameters of MGSTR network 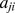 are given in Table 1. This model is an idealization of real gene regulatory networks. Simple though it is, it captures how the topology constrains the dynamics of the gene expression levels and the propagation of information between genes.

**Table 1:** Structure parameter 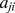 of the MGSTR network.

	miR-17-92	Myc	E2F	p27	pRb	Cdk4/CyclinD	Cdk2/CyclinE	Cdc25A
miR-17-92	–1	1	1	0	0	0	0	0
Myc	0	–1	1	0	0	0	0	0
E2F	–*r*	1	1	0	–*r*	0	0	0
p27	0	0	0	0	0	0	–*r*	0
pRb	0	0	0	0	0	–*r*	–*r*	0
Cdk4/CyclinD	0	0	0	0	0	–1	0	0
Cdk2/CyclinE	0	1	1	–*r*	0	0	0	1
Cdc25A	0	1	1	0	0	0	1	–1

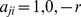
, or 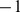 represents respectively the activation, no interaction, inhibition, or self-inhibition between biological molecules.

### 2. Simulation of the Cancer Regulatory Network

By iteratively applying the update rule of Eq. (1), the Boolean network traces a trajectory through the state space. The degradation of biomolecule generally has a time delay, thus 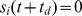 if 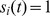 at 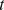. In our simulation, we set the delay time 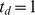 and the time step 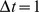. Since the inhibition is often far stronger than activation in the natural biological system, we take 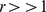 as in references [11] and [23].

It is known that the cellular biochemical reactions occur far from thermodynamic equilibrium, and the copy number of each molecule may be higher or lower than its threshold. Therefore, each node in the network may randomly stay in one of its two states, 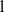 or 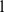. The network’s state is the vector of nodes’ values. Totally, the 8-node MGSTR network will have a state space of 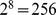 states.

The information processing capacity of a complex dynamic system is reflected in the partitioning of its state space into disjoint basins of attraction. We run the model from each one of the 256 possible states and all nodes are updated simultaneously. It is found that the system dynamic results in five different attractors. The state of attractors and the basin size (B) of each attractor are given in Table 2. It can be seen that most of the states flow into the biggest stationary state attractor or super stable attractor which attracts 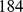 or 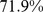 states. It means that, although intrinsic and extrinsic random fluctuations are inevitable, the mammalian G1/S regulatory dynamic pathway is relatively stable, and the MGSTR network is robustly designed.

**Table 2:** Basin size of attraction for the fixed point and network state of each attractor of the MGSTR network.

Basin size	miR-17-92	Myc	E2F	p27	pRb	Cdk4/CyclinD	Cdk2/CyclinE	Cdc25A
184	0	0	0	0	0	0	1	1
48	0	0	0	1	0	0	0	0
16	0	0	0	1	1	0	0	0
6	0	0	0	0	0	0	0	0
2	0	0	0	0	1	0	0	0

**Figure 2:**
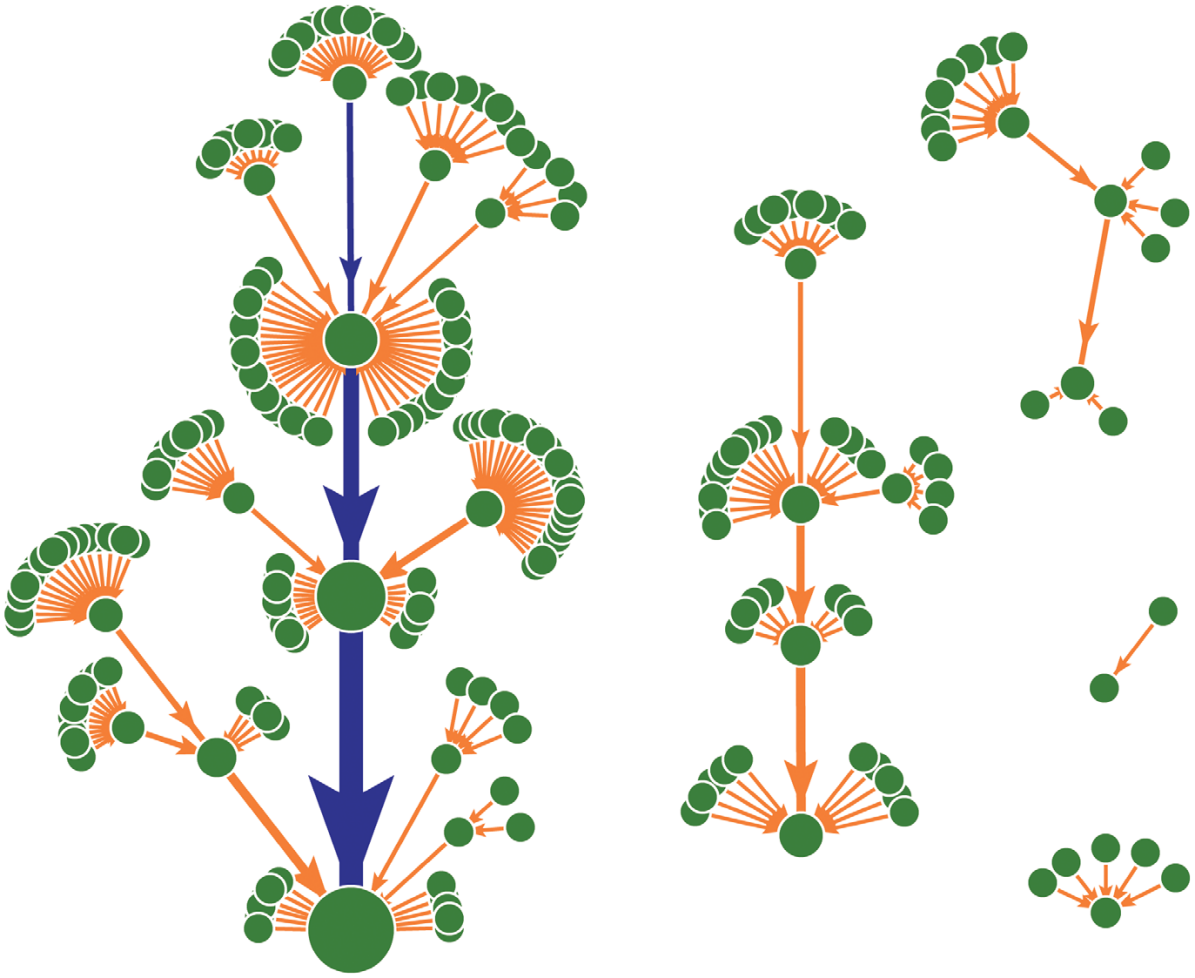
Dynamic trajectories. Dynamic trajectories of the regulatory network with 256 initial states in state space. All states converge towards fixed point attractors. Each green circle corresponds to one specific network state, and the largest circle corresponds to the S phase. Arrows between the network states indicate the dynamic flow from one state to its subsequent state, and the size of flow is indicated by the thickness of arrows.

The state-space graph Fig. 2 provides a visual representation of the system dynamics captured by the state-space analysis. Each green node in this graph represents a Boolean state of the system, and each orange arrow stands for a transition from one state to its temporally subsequent state. The dynamic trajectories of the network and how it converges towards the biggest attractor are shown in Fig. 2. The blue arrows indicate the most possible transition pathway that leads to the biggest attractor.

There are two different interpretations for the function of attractors. One follows Kauffman’s describing that one attractor should correspond to a cell type [24], another interpretation is that they correspond to the cell states of growth, differentiation, and apoptosis [25]. As for our MGSTR network model, the biggest attractor in state space should correspond to the state in which cells overcome the G1/S checkpoint and stay at S phase. In that case, the stability of the cell state is guaranteed.

Previous experimental data demonstrated that the expression or the activation of the key regulators is reflected in the switch characteristics during the G1/S transition. E2F and Myc induce the transcription of miR-17-92 [19], and this miRNA has been shown to suppress the G1/S cell cycle checkpoint by regulating the expression of core genes in cell cycle network [15]. The E2F has high expression levels during G2/M and G0/G1 transition and low expression levels in S phase [26]. The expression of Myc increases in the early G1 restriction point and then returns to a lower level [27]. The expression of Cdc25A phosphatase and the Cdk2/cyclinE kinase are activated by Myc [28]–[30]. The E2F/cyclinE complex appears primarily in the G1 phase, and then its amount decreases as cells enter S phase [26], [30]–[35]. The maximal levels of the p27 protein are found in the G1 phase and quiescence (G0) [7], [28], [30],[36]–[37]. The pRb is phosphorylated in the mid and late G phase, and then the pRb/E2F complex triggers the activation of Cdc25A [7], [27], [32], [35], [38]. The Cdk4/CyclinD or the Cdk6/CyclinD kinase is activated during G1 phase before Cdk2/CyclinE is increased [7],[39]–[40]. G1/S phase transition is regulated by Cdk2/CyclinE [31], [39]–[40]. Activation of Cdc25A occurs during late G1 phase and increases in S and G2 phases [39]. The transitions of above regulators between ON and OFF are summarized in Table 3. A comparison between Table 2 and Table 3 reveals that the biggest attractor is S phase.

**Table 3:** Switch characteristics of key regulators during G1/S transition and the references of corresponding experiments.

	miR-17-92	Myc	E2F	p27	pRb	Cdk4/CyclinD	Cdk2/CyclinE	Cdc25A
G1	OFF/ON	OFF/ON	ON	OFF	ON	ON	OFF/ON	OFF/ON
S	OFF	OFF	OFF	OFF	OFF	OFF	ON	ON
Ref.	[15], [26]	[27]–[30]	[26], [31]–[35]	[7], [28], [30], [36], [37]	[7], [27], [32], [35], [38]	[7], [39], [40]	[31], [39]	[34], [39]

On the other hand, the evolution pathway to the biggest attractor in state space should be convergent onto the potential biological pathway. Is the real or the potential biological pathway in the dynamic trajectories, in other words, how to find out the probable biological pathway in the dynamic trajectories? There is a potential biological pathway to the biggest stationary state attractor (see the thick blue arrow in Fig. 2), and the time sequence of this pathway is listed in Table 4. According to the time sequence in Table 4, there exists four steps for the expression or activation of regulators. Firstly, the expression of E2F, pRb, and CyclinD/Cdk4 is triggered, and the expression of E2F can be activated by itself. The activation of E2F is inhibited by pRb, meanwhile the expression of pRb is inhibited by Cdk4/CyclinD which has a self-degradation effect. Secondly, the expression of miR-17-92, Myc, Cdc25A, and Cdk2/CyclinE is activated by E2F. At the same time, the activation of pRb is inhibited by Cdk2/CylinE. Thirdly, the expression of miR-17-92 is activated by Myc; the expression of Cdc25A is activated by both Myc and Cdk2/CyclinE, and the expression of Cdk2/CylinE is activated by both Myc and Cdc25A. The Myc has a self-inhibition effect. Finally, the expression of Cdc25A and Cdk2/CyclinE can be activated by each other, and the miR-17-92 has a degradation effect. Above results obtained from the mathematical model (Table 4) are consistent with previous experimental results (Table 3).

**Table 4:** The most probable time sequence of the network state that corresponding to the biological pathway, which is indicated by blue arrows in Fig. 2.

Time	miR-17-92	Myc	E2F	p27	pRb	Cdk4/CyclinD	Cdk2/CyclinE	Cdc25A	Phase
1	0	0	1	0	1	1	0	0	G1
2	1	1	0	0	0	0	1	1	G1/S
3	1	0	0	0	0	0	1	1	G1/S
4	0	0	0	0	0	0	1	1	S

### 3. Comparison with Random Network: Robustness Test

**Figure 3:**
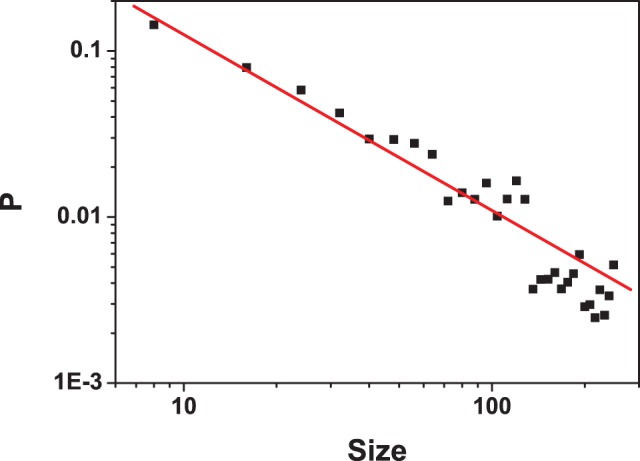
Attractor size distribution of random networks. Calculated from 1000 random networks with the same number of nodes and the same number of lines as our MGSTR network.

To further investigate whether the architecture of this MGSTR network has other special properties, we analyze our network and 1000 random networks with the same number of nodes and the same number of lines as the MGSTR network. It is found that (i) the corresponding random networks typically have more attractors with an average attractor number of 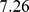. The basin size of the biggest attractor of most random networks is smaller than that of the MGSTR network. This result indicates that attractor basin size of the cancer cell regulatory network is optimized to provide biological function. (ii)The distribution of attractor basin size of these random networks follows a power law (Fig. 3). Only 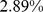 attractors are equal to or larger than the biggest attractor (B = 184) of the MGSTR network.

**Figure 4:**
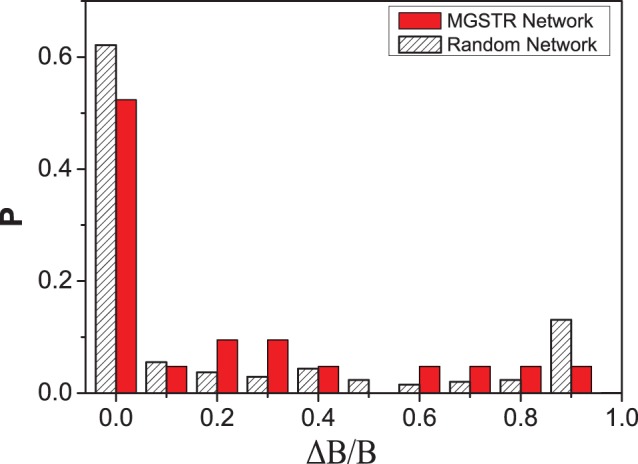
Perturbation of deleting interaction. The distribution of relative changes (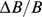) under the perturbation of deleting 21 interaction arrows from the MGSTR network and random networks. The majority of 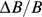 values are small, which indicates that most perturbations will not alter the size of the biggest attractor significantly.

**Figure 5:**
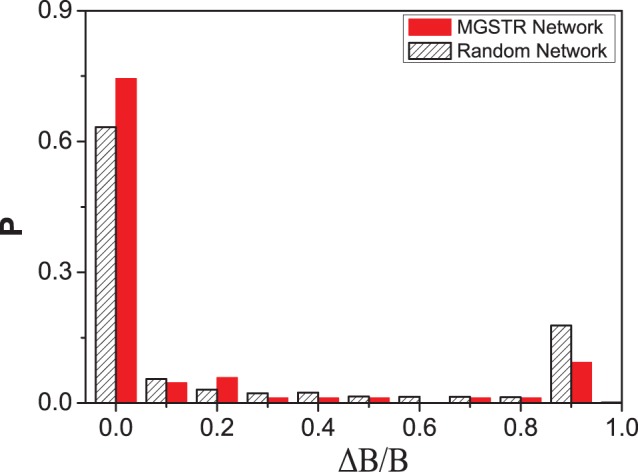
Perturbation of adding interactions. The distribution of relative changes (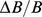) under the perturbation of adding 86 interaction arrows into our MGSTR network. The majority of 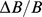 values are small, which indicates that most perturbations will not alter the size of the biggest attractor significantly.

**Figure 6:**
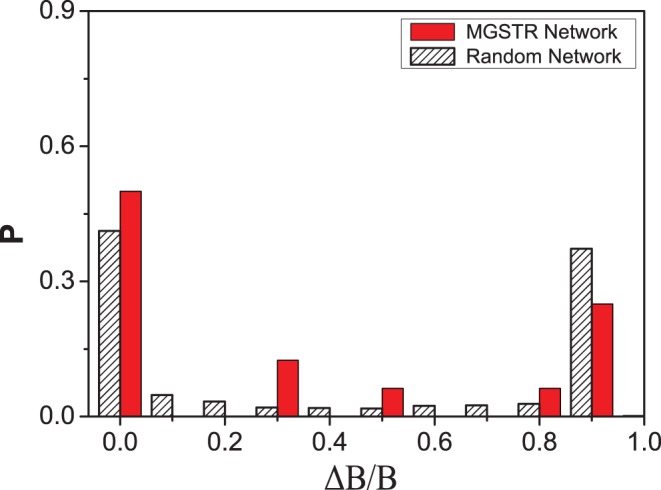
Perturbation of switching interactions. The distribution of relative changes (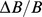) under the perturbation of switching 16 interaction arrows in the MGSTR network. Most of 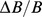 values are small, whereas about 25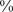 of 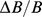 values are located at the interval of 0.9
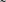
1.0.

The size of basin of attractors (B) in a system is a vital quantity in terms of understanding network behavior and may relate to other network properties such as stability. Therefore, the relative change in B for the biggest attractor 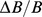 can be served as a measurement in our robustness test. The MGSTR network and the random networks are perturbed by deleting an interaction arrow (Fig. 4), adding a green or blue arrow between nodes that are null-linked (Fig. 5), or switching the interaction of a single arrow from inhibition to activation and vice versa (Fig. 6) [8]. It is shown that most perturbations will not alter the size of the biggest attractor significantly (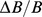 is small)in MGSTR network, which suggests our MGSTR network has high *homeostatic stability* [8]. Such high *homeostatic stability* is not well maintained in the ensemble of random networks with the same size (Fig. 4–6). High robustness of the MGSTR network may be attributed to the structure and interactions within the regulatory system.

### 4. Backbone motif of Cancer Regulatory Network

Given the MGSTR network structure and the time sequence of the pathway which is known to be biologically important, is there a backbone motif that can achieve the major biological functionality? If there is a backbone motif, what is the dynamic behavior of the remaining motif? To address these issues, we adopt the method of process-based network decomposition [11].

**Figure 7:**
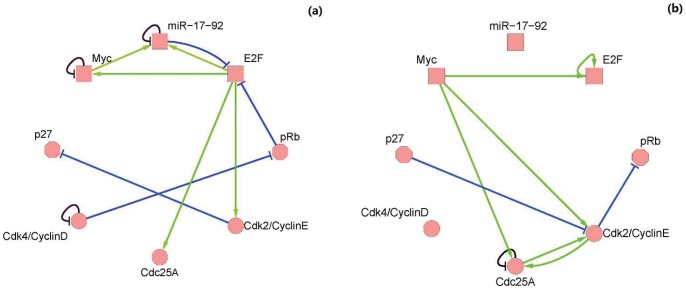
Backbone motif. Full MGSTR network is decomposed into a backbone motif (a) which provides the major biological functions and a remaining motif (b) which makes the system more stable.

For the dynamic function given by Table 4, each node of the network has three logical equations as shown in **Methods**, and solutions of Eqs. (2)–(12) are the minimal lines that should be kept in the construction of backbone motif (Table 5). Basing on Table 5, we extract a backbone motif from the full network as shown in Fig. 7.

**Table 5:** Minimal lines for every nodes of the MGSTR network.

Node ID	Node Name	Minimal Lines
1	miR-17-92	*b*_11_, *g*_21_, *g*_31_
2	Myc	*g*_32_, *b*_22_
3	E2F	*b*_13_, *b*_53_
4	p27	*b*_74_
5	pRb	*b*_65_
6	Cdk4/CyclinD	*b*_66_
7	Cdk2/CyclinE	*g*_37_
8	Cdc25A	*g*_38_

They are obtained by using process-based approach as described in **Methods**.

To investigate the role of backbone motif in the mammalian G1/S regulatory network, we compute the dynamic properties of backbone motif by using the Boolean rule in Eq. (1). The corresponding state of attractors and the basin size from this computation are given in Table 6. It is shown that there are 12 attractors, among which the biggest attractor (the first row in Table 6) corresponds to the super stable attractor of the full network. Therefore, the main function of the MGSTR network is still persisted. The backbone motif is the fundamental building block of the network. However, the basin size of the biggest attractor of the backbone motif is only 120 or 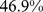 of the initial states, which is much smaller than that of the full network (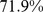). It implies that the remaining part of the network plays important role in real biological regulatory processes, and dynamic properties of backbone motif become unstable without the remaining motif.

**Table 6:** Basin size of attractors and the corresponding network state that acquired from the backbone motif.

Basin size	miR-17-92	Myc	E2F	p27	pRb	Cdk4/CyclinD	Cdk2/CyclinE	Cdc25A
120	0	0	0	0	0	0	1	1
40	0	0	0	0	1	0	1	1
24	0	0	0	0	0	0	1	0
12	0	0	0	0	0	0	0	0
12	0	0	0	0	0	0	0	1
12	0	0	0	1	0	0	0	0
12	0	0	0	1	0	0	0	1
8	0	0	0	0	1	0	1	0
4	0	0	0	0	1	0	0	0
4	0	0	0	0	1	0	0	1
4	0	0	0	1	1	0	0	0
4	0	0	0	1	1	0	0	1

All the interactions between miR-17-92 and other regulatory factors are retained in the backbone motif (Fig. 7). This observation, together with the experimental results in ref. [15], [16], [19], highlights the importance of mir-17-92 in overcoming the G1/S cell cycle checkpoint and increasing the proliferation rate of cancer cells by targeting a network of interacting factors.

## Conclusion and Discussion

Modeling the molecular regulatory network that controls mammalian cell cycle is a challenging and long-term effort. Focusing on the core network that controls the cancer cell cycle, we have constructed a Boolean network with interactions between the oncogenes and tumor suppressor genes (Fig. 1). Although the MGSTR network that we construct is a simplification of intracellular process, study of the relationships between structure and dynamic behaviors of this Boolean network has yielded important insights into the overall behaviors of cancer cell cycle regulatory network. The dynamic of the network is characterized by a dominant attractor in the space of all possible initial states (Fig. 2). It attracts 184 or 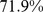 initial states of the Boolean network (Table 2). In addition, based solely on the connection among the nodes, and neglecting other biochemical details, this network reproduces the time sequence of gene activity along the biological cancer cell cycle (biological pathway). The dynamics of our cell cycle network is quite stable and robust for its function with respect to small perturbations (Fig. 4, 5, 6).

There are other cell cycle network models that involve more gene variables than the one we have here. Since the degrees of complexity grow exponentially with the size of the system, it is generally difficult to explore large systems. Recently, various methods have been developed and introduced to investigate the property and the information transition in large Boolean networks. Akutsu et al. presented several algorithms to identify periodic attractors and singleton attractors in Boolean networks [41], [42]. By using gene ordering and feedback vertex sets in the algorithms, Zhang and colleagues identified singleton attractors and small attractors in Boolean networks [43]. Krawitz et al. found that information capacity of a random Boolean network is maximal in the critical boundary between the ordered and disordered phases via introducing a new network parameter, the basin entropy [44].

There usually exists some critical interactions, nodes,or backbone motifs that fulfill the main function in regulatory networks. According to the potential biological pathway in the state space, we further decompose our model into a backbone motif which provides the major biological functions and a remaining motif which makes the system more stable (Table 6). There are other publications that apply various methods to identify important pathways, critical network structures, network motifs, and feedback loops in regulatory networks. For example, Choi et al. constructed a Boolean model of the P53 regulatory network [45]. State-space analysis with an attractor landscape was used to identify specific interactions that were critical for converting cyclic attractors to point attractors in response to DNA damage. The work of Schlatter et al. discussed the discovery of relevant hubs in a network of signaling pathways of apoptosis [25]. Verdicchio et al. recently revealed key players in the network of yeast cell cycle and the network of WNT5A for melanoma by analyzing the logic minimization of the collections of states in Boolean network basins of attraction [46].

The critical role of mir-17-92 in ensuring the checkpoint surpassing in cancer cell cycle is shown in the backbone motif of the MGSTR network (Fig. 7). microRNAs, and more broadly, noncoding RNAs have been increasingly recognized as key regulators in crucial biological events [47]–[51], although roles of the majority of noncoding RNAs still remain elusive. Our work indicates that computational simulation of biological processes may aid future uncovering of regulatory roles of noncoding RNAs.

In our simulation of the MGSTR network, we employ the often used assumption of synchronous update. However, this assumption may be unrealistic in some molecular systems where a variety of timescales, from fractions of one second to hours, are needed to be correctly represented. Some studies modeled and analyzed the asynchronous update rule in the context of random Boolean networks [52], [53]. For example, with synchronous and different asynchronous update methods, Assieh et al. systematically compared the dynamic behaviors displayed by a Boolean network of signal transduction [53]. Their work pointed out that the unperturbed system possesses an update-independent fixed point, while perturbed systems lead to an extended attractor under the disrupting of a particular node. Processes governing gene regulatory networks take place on the molecular level, and fluctuations in the number of molecules of critical factors impact the final output of regulatory networks. Thus, it is highly necessary to apply stochastic simulations for more realistic description of the reaction kinetics. Braunewell et al. investigated the stability of the cell cycle network upon adding a stochastic delay noise [54]. They found that the system exhibits robust behavior under the perturbation of transmission time noise. It would be worth developing our current model to a more realistic one by adding asynchronous update rule and stochastic noise.

Since publication of the seminal work by Kauffman, Boolean network has been one of the most intensively studied models in systems biology [24]. Compared with ordinary differential equation (ODE) models, Boolean networks are limited in approximating experimental results and in making context-specific quantitative predictions of cellular dynamics. However, applications of Boolean network in modeling real biological circuits have shown that they can predict consequences of protein and gene activities with much fewer parameters than the classical differential equations. Our results from the analysis of the MGSTR network demonstrate that Boolean model can be used to simulate cancer G1/S cell cycle process.

## Materials and Methods

### Decomposition of Regulatory Network

Let 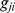 and 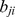 represent respectively activated line and inhibitory line. The values of 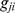 and 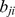 are 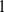 or 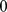, which represents exist or no exist, respectively. Then, one can obtain a logical equation for each node from Eq. (1):

(2)where the operational symbols are logic symbols: addition represents operator OR, multiplication represents operator AND, and bar represents NOT.

With the four possible transitions of state 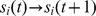, Eq. (2) can be replaced by
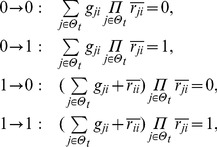
(3)where 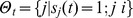. The first and third equations in Eq. (3) can be converted by performing NOT on both sides, thus



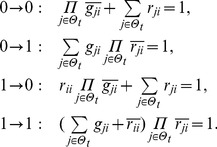
(4)For each node, there are 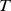 transitions, and thus there are 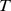 equations since 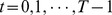. With the time sequence shown in Table 4, from Eq. (4), we have
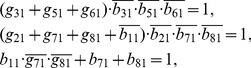
(5)for node 1 (node name: miR-17-92);
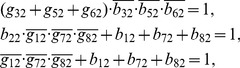
(6)for node 2 (node name: Myc);
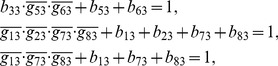
(7)for node 3 (node name: E2F);
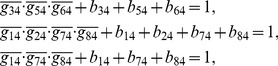
(8)for node 4 (node name: p27);
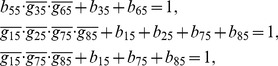
(9)for node 5 (node name: pRb);
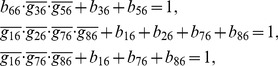
(10)for node 6 (node name: Cdk4/CyclinD);
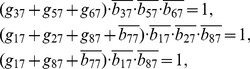
(11)for node 7 (node name: Cdk2/CyclinE);
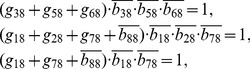
(12)for node 8 (node name: Cdc25A).

The networks in this paper were drawn with Cytoscape [55], [56] and the dynamical state space graph was drawn with Pajek [57].
